# Effect of Substituent Groups on the Strength of Intramolecular Hydrogen Bonds in 2,4-Dihydroxybenzophenone UV Absorbers

**DOI:** 10.3390/molecules28135017

**Published:** 2023-06-27

**Authors:** Zhengjun Fang, Xinhua Zhang, Feng Wu, Baoyu Huang, Chaktong Au, Bing Yi

**Affiliations:** Hunan Provincial Key Laboratory of Environmental Catalysis & Waste Recycling, College of Materials and Chemical Engineering, Hunan Institute of Engineering, Xiangtan 411104, China; zxh1398043146@163.com (X.Z.); 70223@hnie.edu.cn (F.W.); pctowl@hotmail.com (C.A.)

**Keywords:** substituents, 2,4-dihydroxybenzophenone, density functional theory, hydrogen bonding

## Abstract

2,4-Dihydroxybenzophenone is the most widely used molecule in the benzophenone group of UV absorbers. It is known that the UV absorption ability is dependent on the substituents. Numerous studies have shown that the strength of intramolecular hydrogen bonds is the main factor affecting this type of UV absorber. However, the effect of substituents on the formation and nature of the hydrogen bonds has not been well studied. In this work, the effect of the type of substituent and the substitution position on the absorption intensity of 2,4-dihydroxybenzophenone molecules is verified both experimentally and theoretically. The effect of substituents on the intramolecular hydrogen bonding of 2,4-dihydroxybenzophenone was investigated by DFT calculations. The results indicate that the addition of different substituents leads to various changes in the strength of the hydrogen bonding in 2,4-dihydroxybenzophenone. On the X-substitution site or the Y-substitution site, halogen groups and electron-absorbing groups such as -CN and -NO_2_ increase the strength of the hydrogen bond, while electron-giving groups such as -N(CH_3_)_2_ and -OCH_3_ decrease the strength of the bond. For the same substituent, the one at the Y site has a higher effect on hydrogen bonding than that at the X site. By NBO analysis, it was found that the substituents would cause charge redistribution of the individual atoms of 2,4-dihydroxybenzophenones, thus affecting the formation and strength of the hydrogen bonds. Moreover, when the substituent is at the Y substitution site, the oxygen atom of the carbonyl group is less able to absorb electrons and more charge is attracted to the oxygen atom of the hydroxyl group, resulting in a larger charge difference between the two oxygen atoms and an increase of bond energy. Finally, a multiple linear regression analysis of the NPA charge number of the atoms involved in the formation of the hydrogen-bonded chelated six-membered ring was performed with the energy of the hydrogen bond and the percentage of influencing factors estimated, which were found to jointly affect the strength of hydrogen bonding. The aim of this study is to provide theoretical guidance for the design of benzophenone-based UV absorbers that absorb UV light of specific wavelength bands.

## 1. Introduction

UV rays that reach the Earth’s surface through the atmosphere can cause skin damage and even skin cancer, and UV resistance has become an important indicator of the functionality of textiles. Textiles with good UV resistance are widely used and have good market prospects. With wavelengths of 200–400 nm, ultraviolet light is divided into short-wave UV, medium-wave UV, and long-wave UV. Short-wave UV (UV-C, 200–280 nm) is absorbed by the ozone layer in the atmosphere, and about 10% of medium-wave UV (UV-B, 280–320 nm) and 90% of long-wave UV (UV-A, 320–400 nm) penetrate the atmosphere and reach the Earth’s surface [1]. The UV absorbers prevent direct exposure of the skin to UV light, usually by absorbing UV light and converting the photonic energy to other forms of energy such as heat. Several common UV absorbers are benzophenone derivatives, for example, benzotriazoles, substituted acrylonitriles, triazines, hindered amines, and salicylates [2,3,4,5].

Benzophenones are the most common and inexpensive type of UV absorbers used in a wide range of textiles [6,7,8,9,10]. Among them, 2,4-dihydroxybenzophenone (UV-0) is the simplest, and modification of it or the introduction of different substituents can induce different effects on the UV absorber. By reacting carboxymethyl chitosan (CMC) hydrochloric acid with UV-0, Yu et al. [11] produced UV-0 absorbers with increased absorption capacity. By reacting benzoic acid with resorcinol and then sulfonating the acylated product, Cao et al. [12] synthesized the UV absorber 2,4-dihydroxy-5-sulfonicacidbenzophenone, which was found to have good water solubility. Furthermore, from UV-0 and chlorooctane, Tong et al. [13] generated 2-hydroxy-4-n-octanoxy dibenzophenone with improved thermal stability.

UV-0 works as an absorber by forming an intramolecular hydrogen bonded chelate ring using its carbonyl group and nearby hydroxyl group. The absorption of UV energy would result in the breaking of the intramolecular hydrogen bond, and with the opening of the chelate ring, there is the conversion of radiation energy to thermal energy. In addition, the carbonyl group in the molecule is excited by the absorbed UV energy, leading to reciprocal isomerization, which is also energy consuming [14,15,16]. Therefore, in the absorption of UV radiation by benzophenones, the intramolecular hydrogen bonds play an important role. Hydrogen bonds are weak interactions (essentially due to electrostatic effect) which are formed by the mutual attraction between a H atom connected to an atom of high polarity and the lone pair electrons of another highly electronegative atom, leading to change of molecule conformation and extension of conjugate structure. Consequently, the absorption spectrum and maximum absorption wavelength of the molecule is altered, together with the absorption properties of the absorber adjusted [17]. Tan et al. [18] investigated the effect of substituents on the performance of UV absorbers that were based on benzophenone derivatives and observed enhanced absorption performance of UV-0. Zahedi-Tabrizi et al. [19,20] conducted a theoretical study on the effect of substituents on the intramolecular hydrogen bonding of 2-hydroxy benzophenone using Cl as substituent at various positions. However, none of these studies investigated the effect of substituents on the formation and strength of hydrogen bonds at a molecular level.

With this background, we proceeded to verify the effect of substituents on the UV absorption intensity of 2,4-dihydroxybenzophenones through a combination of experimental and theoretical studies. The effect of the type and position of substituent on the intramolecular hydrogen bonding of 2,4-dihydroxybenzophenone was then investigated using DFT. The percentage of influencing factors was analyzed to relate the hydrogen bonding energy with the NPA charge number of the atoms involved in the formation of the hydrogen-bonded chelated six-membered ring. According to Reed et al., as an alternative to traditional Mulliken layout analysis, natural population analysis (NPA) seemed to better describe the electron distribution in the compounds of high ionic character and better numerical stability [21].

## 2. Results and Discussion

### 2.1. UV Absorption Performance of 2,4-Dihydroxybenzophenone Absorbers

**Figure 1:**
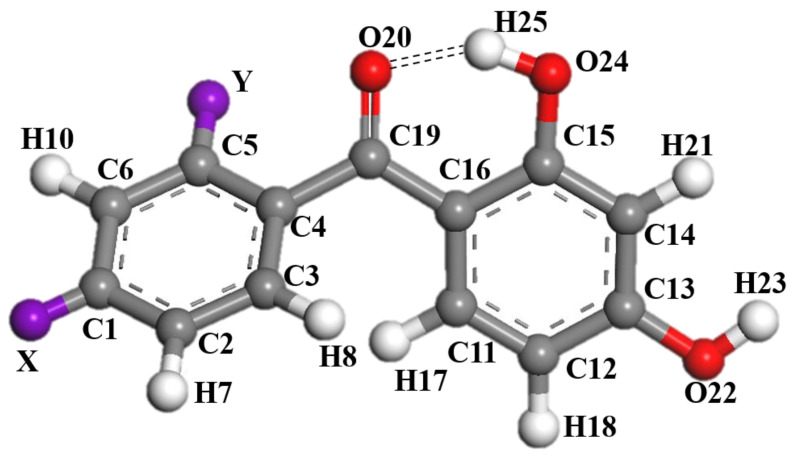
The stable structure and numbering systems of 2,4-dihydroxybenzophenone.

Figure 1 gives the structure and numbering system of the 2,4-dihydroxybenzophenone (2,4-DBH) class of UV absorbers, where X and Y are substituent sites. At each of the sites, fourteen substituents (-H, -F, -Cl, -Br, -OCH_3_, -OCH_2_CH_3_, -CH_3_, -CH_2_CH_3_, -C(CH_3_)_3_, -CN, -NH_2_, -NO_2_, -N(CH_3_)_2_, -COCCH_2_CH_3_, and -OH.) are analyzed. Substitution is of one substituent at a time. All the molecules optimized for stable structure involved in this study are shown in Figure S1.

**Figure 2:**
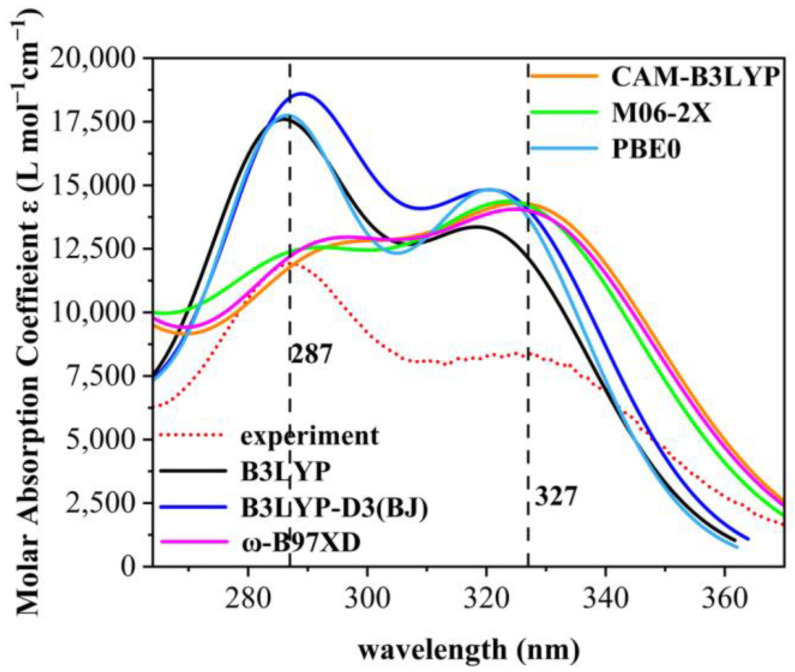
Experimental spectra of 2,4-DBH and comparison of six calculation methods. The red dashed line is the plot obtained from experimental measurements. UV spectra of the first 70 excited states of 2,4-dihydroxybenzophenone in ethanol solvent were calculated for B3LYP, B3LYP-D3(BJ), CAM-B3LYP, M06-2X, PBE0, and ω-B97XD under the def2-TZVPP group.

As only medium- and long-wave ultraviolet light can reach the Earth’s surface, the paper addresses the ultraviolet spectrum in the 280–380 nm range. As shown in Figure 2, the UV absorption spectra were calculated using six different general functions and at the same line-broadening, it was found that the absorption spectra calculated using the B3LYP, B3LYP-D3, and PBE0 general functions had only single absorption peaks, whereas those using CAM-B3LYP, M06-2X, and ω-B97XD all had double absorption peaks. The absorption spectra of the other eight are shown in Figure S3. To demonstrate the reliability of the theoretical calculations, the UV absorption spectra of the nine organic compounds were measured experimentally. Standard cuvettes were washed with anhydrous ethanol, then 2.0 × 10^−4^ g of each compound was introduced into a centrifuge tube (numbered sequentially) and diluted with anhydrous ethanol. The measurement was conducted using anhydrous ethanol as reference solution employing a UV meter. The UV meter was a Shimadzu UV-1800 UV Spectrophotometer from Octagon Scientific Instruments Ltd. (Changsha, China). The maximum absorption wavelength of the experimental ultraviolet absorption spectra of 9 UV absorber molecules are depicted in Table S1. The experimental ultraviolet absorption spectra of the nine organic compounds are shown in Figure S2.

Comparing the spectral data of the six generalized calculations with that of the experimentally measured data, we found that the spectra from the M06-2X generalized calculations are in high agreement with the experimental data, which indicates that all the results of theoretical calculations are highly accurate [22,23]. The comparison of experimental and theoretical spectra of the nine molecules shows that the M06-2X generalization approach can calculate UV maps in good agreement with the experimental measurements, and therefore the M06-2X generalization method was used in the subsequent UV calculations of organic molecules in the present study.

**Figure 3:**
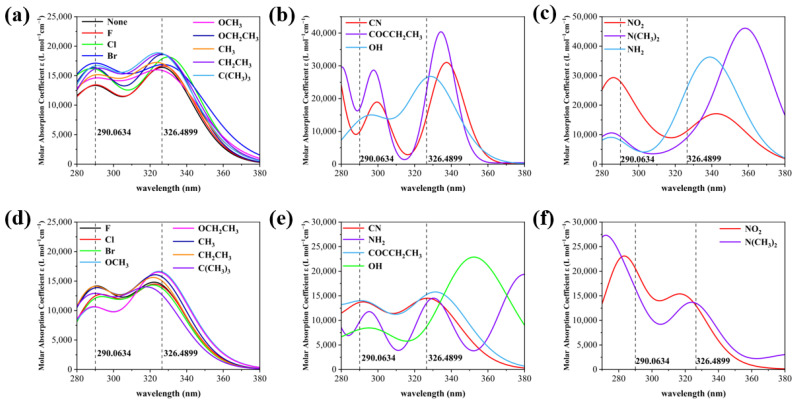
(**a**) UV spectra of nine molecules at the X site at which experimental spectra were measured, (**b**) UV spectrum of a molecule with a significant red shift in the wavelength of the two absorption peaks of 2,4-DBH at the X site, (**c**) UV spectrum of a molecule with a significant blue shift in the wavelength of the two absorption peaks of 2,4-dihydroxybenzophenone at the X site, (**d**) UV spectrum of a molecule with the same substituent as in (**a**) at position Y, (**e**) UV spectrum of a molecule with a significant red shift in the wavelength of the two absorption peaks of 2,4-DBH at the Y position, (**f**) UV spectrum of a molecule with a significant blue shift in the wavelength of the two absorption peaks of 2,4-DBH at the Y position.

As shown in Figure 3, the UV absorption spectra of the X substituents are classified into three categories based on experimental spectral comparison and the obvious red and blue shifts, and the Y substituents are also classified into three categories for comparison between X and Y substitution. Compared to the wavelength corresponding to the peak of the maximum absorption of 2,4-DBH (the left absorption peak has a wavelength of 290.0634 and the right absorption peak has a wavelength of 326.4899), For the electron-absorbing groups such as -CN, -NO_2_, -F, -Cl, and -Br, the X-substitution position shows a blue shift in the left absorption peak and a gradual red shift in the right absorption peak, whereas for the electron-giving groups such as -OCH_3_, -OCH_2_CH_3_, and -N(CH_3_)_2_, the left absorption peak and right absorption peak show a red and blue shift, respectively. The electron-absorbing groups at the Y-substituted position have an overall tendency to red-shift the absorption peak on the left and to blue-shift the absorption peak on the right. The absorption peaks at both X and Y substitution positions are generated by π→π* transitions. Most of the absorption peaks on the left side are generated by the S0→S3 excitation transition, and most of the peaks on the right side are generated by the S0→S2 excitation transition, the specific contributions of the transitions to the absorption peaks are shown in Table S2.

### 2.2. Effect of Substituent Groups on the Strength of Intramolecular Hydrogen Bonds in 2,4-Dihydroxybenzophenone Absorbers

Intramolecular hydrogen bonding plays a central role in the absorption of UV radiation by benzophenone UV absorbers. Hydrogen bonds are weak interactions, essentially an electrostatic effect, which is a result of mutual attraction between a H atom connected to an atom of high polarity and the lone pair electrons of another atom of very high electronegativity. Consequently, there is not only a change of molecular conformation but also a lengthening of conjugated structure, thus affecting the absorption spectrum and maximum absorption wavelength of the molecule [17]. To understand the effect of substituents on the absorption performance of the absorber, it is necessary to analyze the strength of intramolecular hydrogen bonding in the 2,4-dihydroxybenzophenones absorbers.

There are many researchers who have analyzed the nature of hydrogen bonding and estimate the strength of hydrogen bonds based on the “bond critical point” properties defined by the atoms-in-molecules (AIM) theory. According to the theory, a bond critical point (BCP) exists between two interacting atoms in close proximity, and the total curvature of the electron density (ρ-BCP) at this point is the electron density Laplace value (∇^2^_ρ-BCP_). It is often used as a method to determine the type of interaction between two atoms. A negative sign is indicative of covalent interaction with sharing of electron pairs between the atoms, and bond formation is where the electron density is the highest. In the case of a positive sign, the bonding is considered to be a closed-shell interaction, such as hydrogen bonding, ionic bonding, and pi-pi stacking, which are electrostatic or van der Waals in nature, so there is no concentration of electron density [19]. The position in a molecule where the electron density reaches a maximum at a point on the ring is called the ring critical point electron density (RCP), which is an important parameter for describing the ring structure formed as a result of hydrogen bonding.

Subject to AIM analysis, the ∇^2^_ρ-BCP_ of the 29 2,4-dihydroxybenzophenone absorber molecules were calculated (Table 1). The Laplace electron density values are all positive, which is characteristic of closed-shell interactions, indicative of electrostatic or van der Waals interaction. This verifies the essence of hydrogen bonds. RCP and ring-critical point electron density Laplacian values (∇^2^_ρ-RCP_) were calculated for twenty-nine 2,4-Dihydroxybenzophenone absorbers molecules. The hydrogen bond energy (E_O···H_) was calculated based on the electron density of BCP in kcal/mol [24], and the electron density ρ(BCP) at BCP in a.u. Table 1 depicts the bond energy (E_O···H_) and bond length (d_O···H_) of the 29 compounds.

E_O···H_ = −223.08 × _ρ-BCP_ + 0.7423

**Figure 4:**
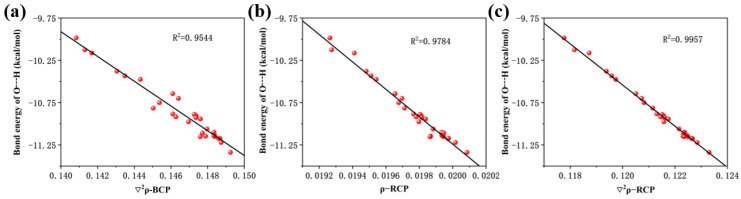
(**a**) Linear relationship between the Laplace value of the electron density at the critical point of the bond and the bond energy of the hydrogen bonds, (**b**) linear relationship between electron density at the critical point of the ring and the bond energy of the hydrogen bonds, and (**c**) linear relationship between the Laplace value of the electron density at the critical point of the ring and the bond energy of the hydrogen bonds.

We performed scatter plots of hydrogen bonding energies against bond critical point electron density Laplacian, ring critical point electron density, and ring critical point electron density Laplacian and found good correlation, as shown in Figure 4a–c.

**Figure 5:**
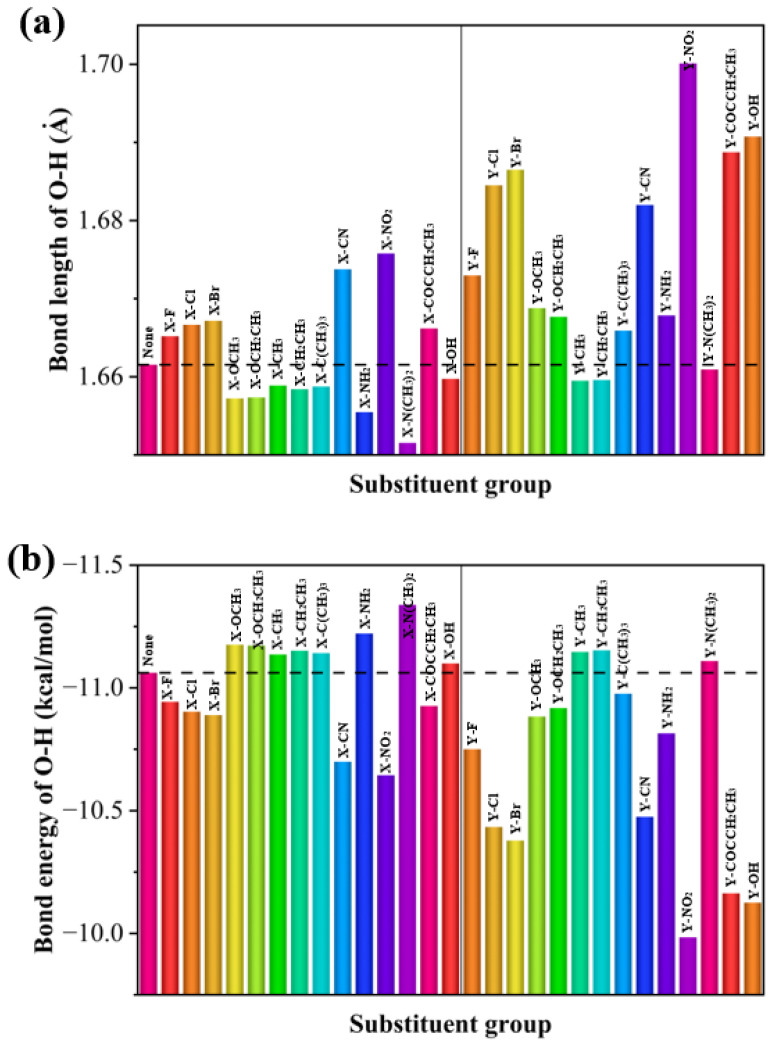
(**a**) Hydrogen bond lengths and (**b**) hydrogen bond energies of 29 molecules.

To analyze the effect of substituents on the strength of hydrogen bonds, bar plots of the bond lengths and bond energies of the intramolecular hydrogen bonds with respect to the substituents were made. The effect of different substituents on hydrogen bonding varies and the effect of the substituent is dependent on the position of the substitution. As can be seen from Figure 5, the hydrogen bonding energy of the substituents is higher at the Y position than that at the X position, and the halogen groups, as well as -CN and -NO_2_, increase the intramolecular hydrogen bonding energy at both the X and Y positions. For the halogen, -CN, and -NO_2_ electron-absorbing groups, the electron-absorbing effect strengthens hydrogen bonding. The hydrogen bonding energy gradually increases as the radius of the halogen group atom increases. However, electron-giving groups such as -OCH_3_ and -N(CH_3_)_2_ reduce the energy of hydrogen bonding.

**Figure 6:**
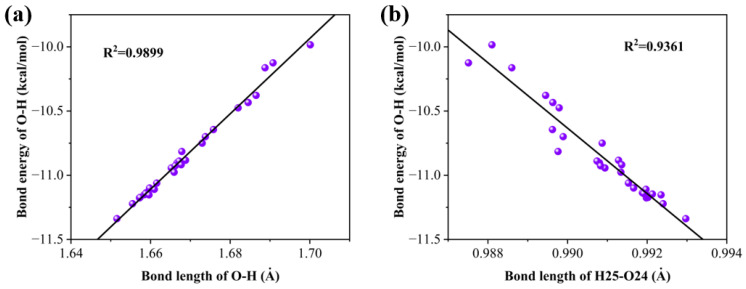
(**a**) Linear relationship between bond energy and bond length for hydrogen bonds and (**b**) linear relationship between hydrogen bond energy and bond length of the bond formed by H25–O24.

We calculated the relationship between hydrogen bond energy and bond length, as well as the relationship between the hydrogen bond energy and the bond length of H25–O24. As shown in Figure 6, it is found that in both cases, there is a good linear relationship, showing that the energy of a hydrogen bond is also influenced by the hydroxyl group that forms the hydrogen bond.

In the combined analysis of the hydrogen bonding energy of nine X-positioned 2,4-dihydroxybenzophenone absorbers with UV resistance, it was found that -Br is the smallest in hydrogen bond energy and the largest in hydrogen bond length, and therefore it should be the worst in UV resistance. Among the remaining eight molecules, -CH_2_CH_3_ is the shortest in hydrogen bond length and thus largest in hydrogen bond energy, and should therefore have the best UV absorption properties. Of the molecules substituted with halogen groups, the hydrogen bond energies decrease in the order of -F > -Cl > -Br, so -F should have the best UV resistance. Overall, the results of theoretical analysis are consistent with the experimental results.

Of the molecules substituted with alkoxy groups, the hydrogen bond energy of -OCH_3_ (containing methoxy) is smaller than that of -OCH_2_CH_3_ (containing ethoxy), so the UV resistance of -OCH_2_CH_3_ should be higher than that of -OCH_3_, but because the hydrogen bonding energies of the two are relatively close, a calculation error is considered possible, leading to a situation where -OCH_3_ has a higher UV absorption capacity than -OCH_2_CH_3_, which is generally consistent with the experimental observation.

Among -CH_3_, -CH_2_CH_3_, and -C(CH_3_)_3_ with alkyl substitution, they are similar in hydrogen bond energy, with -CH_3_ being slightly higher, so theoretically -C(CH_3_)_3_ has a higher UV resistance than -CH_2_CH_3_ and -CH_3_, with -CH_3_ being the worst. Experimentally, when on polyester, -CH_2_CH_3_ did have greater UV resistance than -C(CH_3_)_3_, while the UV resistance of -CH_3_ is the lowest.

By studying the effect of substituents on the hydrogen bond strength of 2,4-dihydroxybenzophenones, it was found that substituents with strong electron-donating power (e.g., -OCH_3_) strengthen the intramolecular hydrogen bonding and increase the UV absorbing capacity, while those with electron-withdrawing power (e.g., -Br) weaken the intramolecular hydrogen bond and decrease the UV absorbing capacity. The presence of an unsaturated bonding group increases the strength of the hydrogen bond due to its electron-absorbing nature.

### 2.3. Electrostatic Potential and Charge Analysis of 2,4-Dihydroxybenzophenone Molecules

**Figure 7:**
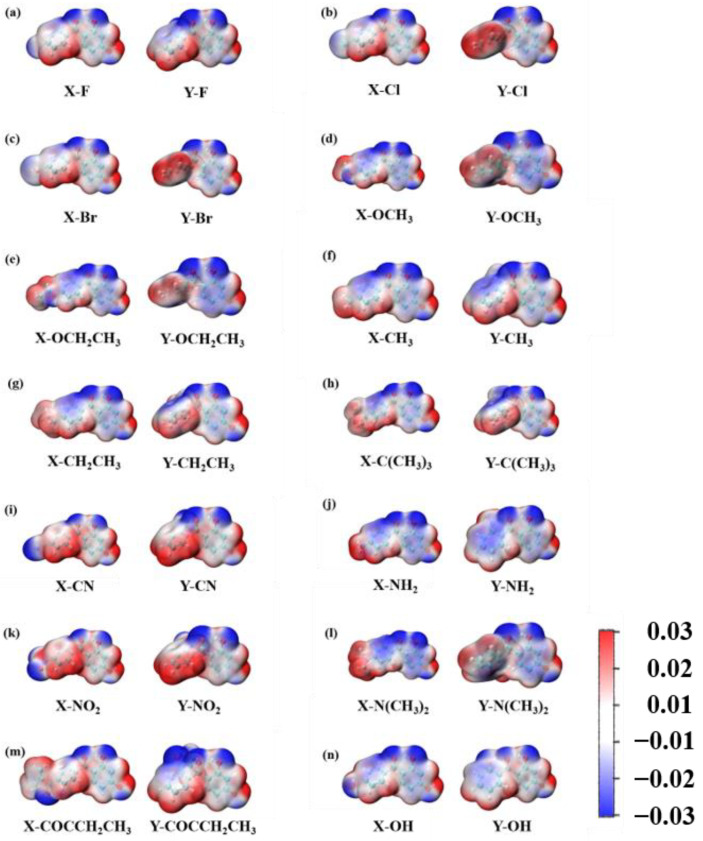
(**a**–**n**) shows the electrostatic potential of 2,4-DBH at the X and Y substitution position. The units are a.u.

As displayed in Figure 7, the 29 2,4-dihydroxybenzophenone molecules were analyzed for their electrostatic potential (ESP), the positivity and negativity of which give some indication of the electrical distribution of the molecules. The red part represents the area of positive concentration and the blue part the area of negative concentration. The results reveal that the 29 2,4-dihydroxybenzophenone absorbers show negative electrical properties around the carbonyl oxygen atom (O20) and positive electrical properties around the hydrogen atom on the hydroxyl group (H25). Therefore, it can be assumed that the carbonyl oxygen atom and the hydroxyl hydrogen atom are the acceptor and donor, respectively, for the formation of hydrogen bonds within the molecules. Moreover, the electronegativity of the O20 atom at the X substitution site is greater than that at the Y substitution site except for -OH.

Substituent groups are important structural units of benzophenone-based UV absorbers, which can affect the formation and strength of the hydrogen bonds by altering the charge distribution within the absorber molecule. We analyzed the atomic NPA charge using NBO as a means to investigate the effect of the substituent group on the formation and bond strength of the intramolecular hydrogen bond of 2,4-dihydroxybenzophenone. The charges of the six atoms of the intramolecular hydrogen-bonded chelate ring are listed in Table 2: q(H25) is the charge of the hydrogen atom involved in the formation of the hydrogen bond, q(O20) is the charge of the oxygen atom involved in the formation of the hydrogen bond, q(O24) is the charge of the oxygen atom attached to the hydrogen atom involved in the formation of the hydrogen bond, q(C15) is the charge of carbon atom 15, q(C16) is the charge of carbon atom 16, and q(C19) is the charge of carbon atom 19, Δ(q(O20) − q(O24)) is the difference in charge between q(O20) and q(O24).

By comparison, it was found that the NPA charge of the atoms involved in the formation of the hydrogen-bonded chelated six-membered ring in the substituted 2,4-dihydroxybenzophenone either increased or decreased compared to that of the atoms in unsubstituted 2,4-dihydroxybenzophenone, suggesting that the substitution of the substituent caused charge redistribution of the individual atoms of 2,4-dihydroxybenzophenone, thus affecting the formation and strength of the hydrogen bond. The charge of the substituent at position Y of q(O20) was found to be lower than that of the substituent at position X for the same substituent. Under the same substituent conditions Δ(q(O20) − q(O24)) at the Y position is greater than that at X position, which means that the oxygen atom of the carbonyl group is less able to absorb electrons. This is because when the substituent is at Y, more charges are attracted to the oxygen atom of the hydroxyl group, making the difference larger and increasing the bond energy.

A regression analysis of the charge number of individual atoms against the hydrogen bond energy reveals that there is no linear relationship between the two (Figure S5). This demonstrates that the hydrogen bond energy is not influenced by the individual atoms, but rather by the atoms of the chelated six-membered ring that form the hydrogen bond.

Therefore, a regression analysis of the hydrogen bond energy E_O···H_ and the charges of the six atoms was done and a multiple linear regression equation was obtained:

E_O···H_ = 0.50333q(H25) − 0.62297q(O20) − 0.66924q(O24) − 0.4073q(C15) − 0.24986q(C16) + 0.54071q(C19) + 0.40726

R2 is 0.847, demonstrating a good degree of regression. An analysis of the percentage of influencing factors was done, giving a descending order of q(O24) > q(C16) > q(O20) > q(C15) > q(C19) > q(H25). The result indicates that the charges of the six atoms together influence the magnitude of the hydrogen bond energy. In other words, the charges of the oxygen atoms of the hydroxyl group, the carbon atoms in the middle, the oxygen atoms involved in the formation of the hydrogen bond, and the hydrogen atoms involved in the formation of the hydrogen bond are important factors affecting the hydrogen bond energy.

## 3. Computational Method

This paper follows the usual approach of quantum chemical calculation, using theoretical methods of various accuracy for different computational tasks. All relativistic density functional theory (DFT) computations were performed using the Gaussian16 software package [25]. The compound geometries were optimized by B3LYP hybrid density functional theory and all atoms (H, C, O, N, F, Cl, Br) were used in 6-311+G(d, p) basis with dispersion functions [26]. The vibrational frequency calculations were kept at the same calculation level for the optimization calculations and the results showed no imaginary frequencies, indicating that the optimized molecule structure is stable. The single point energy calculations for the system were performed at the B3LYP/def2-TZVPP level. In the calculation of the optimized molecular structure, 6-311+G(d, p) was used while in the calculation of the single point energies and spectra, we used the more accurate def2-TZVPP. Non-covalent interactions were considered by the D3(0) correction. Atoms-in-molecules analysis (AIM) [27] and natural bond orbital (NBO) [28] analysis were performed by Multiwfn 3.8 [29]. The correlation wave function analysis removed the dispersion function from the ma-Def2-svp basis set as required by the Multiwfn manual. In addition, UV–Vis spectrograms were adopted to calculate the first 70 excited states in an ethanol solvent using the widely used general functions (i.e., B3LYP, B3LYP-D3(BJ) [30,31], CAM-B3LYP [32], M06-2X [33], PBE0 [34], and ω-B97XD [35]) under the same basic group conditions.

## 4. Conclusions

The effect of substituent type and substitution position on the absorption intensity of 2,4-dihydroxybenzophenone molecules was verified on the basis of experimental results and theoretical calculations of UV spectra. Compared with the UV absorption spectrum of 2,4-dihydroxybenzophenone, there is a blue shift in the left absorption peak of the X-substituted group and a gradual red shift in the right absorption peak, and a red shift in the left absorption peak and a blue shift in the right absorption peak of the electron-donating group. The electron-absorbing group at the Y position has an overall tendency to red-shift the absorption peak on the left and blue-shift the absorption peak on the right, giving a clear blue-shift to the absorption peak on the right of the electron group. DFT calculations, electronic structure, and property analysis were then used to investigate the effect of substituents and the substitution position on the intramolecular hydrogen bonding of 2,4-dihydroxybenzophenone. The results show that the addition of different substituents causes different changes in the strength of the hydrogen bond of 2,4-dihydroxybenzophenone, and the position of the substituent has different effects on the strength of the hydrogen bond. The halogen group and electron-absorbing groups such as -CN and -NO_2_ increase the strength of the hydrogen bond and the electron-giving groups -N(CH_3_)_2_ and -OCH_3_ decrease the strength of the hydrogen bond at both the X and Y positions. The same substituent at Y site has a better effect on hydrogen bonding than that at X site. The NBO analysis revealed that the substituents affect the formation and strength of intramolecular hydrogen bonding by redistributing the charges of the individual atoms of 2,4-dihydroxybenzophenones. In addition, when the substituent is at Y position, the oxygen atom of the carbonyl group is less able to absorb electrons and more charge is attracted to the oxygen atom of the hydroxyl group, resulting in a greater difference in charge between the two oxygen atoms and an increase in bond energy. Finally, a multiple linear regression analysis and an analysis of the percentage of influencing factors between the NPA charge of the atoms involved in the formation of the hydrogen bonded chelated hexameric ring and hydrogen bond energy were performed. It was disclosed that they collectively influence the hydrogen bonding. The results of this study provide theoretical guidance for the molecular design of benzophenones as UV absorbers in specific wavelength bands.

## Figures and Tables

**Table 1:** Bond critical point Laplace electron density values, ring critical point electron density, ring critical point Laplace electron density values, hydrogen bond lengths, bond energies, and H25 and O24 bond lengths of 2,4-dihydroxybenzophenone molecules.

Substituent	∇^2^ρ_-BCP_ (a.u.)	ρ_-RCP_ (a.u.)	∇^2^ρ_-RCP_ (a.u.)	E_O···H_ (kcal/mol)	d_O···H_ (Å)	d_H25–O24_ (Å)
None	0.1480	0.0199	0.1221	−11.0613	1.6615	0.9915
X-F	0.1476	0.0198	0.1217	−10.9434	1.6652	0.9909
X-Cl	0.1474	0.0198	0.1216	−10.9032	1.6667	0.9908
X-Br	0.1473	0.0198	0.1215	−10.8890	1.6672	0.9907
X-OCH_3_	0.1487	0.0200	0.1227	−11.1760	1.6572	0.9920
X-OCH_2_CH_3_	0.1486	0.0200	0.1226	−11.1724	1.6574	0.9920
X-CH_3_	0.1484	0.0199	0.1224	−11.1363	1.6589	0.9919
X-CH_2_CH_3_	0.1484	0.0199	0.1225	−11.1515	1.6584	0.9920
X-C(CH_3_)_3_	0.1483	0.0199	0.1225	−11.1418	1.6588	0.9919
X-CN	0.1464	0.0197	0.1207	−10.6994	1.6738	0.9899
X-NH_2_	0.1487	0.0200	0.1228	−11.2216	1.6555	0.9924
X-NO_2_	0.1461	0.0197	0.1205	−10.6443	1.6758	0.9896
X-N(CH_3_)_2_	0.1492	0.0201	0.1233	−11.3382	1.6515	0.9930
X-COCCH_2_CH_3_	0.1474	0.0198	0.1216	−10.9266	1.6662	0.9908
X-OH	0.1483	0.0199	0.1224	−11.0994	1.6597	0.9917
Y-F	0.1454	0.0197	0.1208	−10.7501	1.6730	0.9909
Y-Cl	0.1435	0.0195	0.1196	−10.4334	1.6845	0.9896
Y-Br	0.1431	0.0195	0.1194	−10.3781	1.6865	0.9895
Y-OCH_3_	0.1461	0.0198	0.1213	−10.8832	1.6688	0.9913
Y-OCH_2_CH_3_	0.1463	0.0198	0.1215	−10.9179	1.6677	0.9914
Y-CH_3_	0.1479	0.0199	0.1223	−11.1465	1.6595	0.9921
Y-CH_2_CH_3_	0.1476	0.0199	0.1223	−11.1529	1.6596	0.9924
Y-C(CH_3_)_3_	0.1470	0.0198	0.1216	−10.9763	1.6659	0.9913
Y-CN	0.1443	0.0195	0.1197	−10.4752	1.6820	0.9898
Y-NH_2_	0.1450	0.0197	0.1211	−10.8148	1.6679	0.9898
Y-NO_2_	0.1408	0.0193	0.1178	−9.9842	1.7001	0.9881
Y-N(CH_3_)_2_	0.1477	0.0199	0.1223	−11.1098	1.6609	0.9920
Y-COCCH_2_CH_3_	0.1417	0.0194	0.1187	−10.1631	1.6888	0.9886
Y-OH	0.1413	0.0193	0.1182	−10.1251	1.6908	0.9875

**Table 2:** NPA charges of the six atoms involved in the formation of the hydrogen-bonded chelated six-membered ring.

Substituent	q(H25)	q(O20)	q(O24)	q(C15)	q(C16)	q(C19)	Δ(q(O20) − q(O24))
None	0.5033	−0.6230	−0.6682	0.4073	−0.2499	0.5407	0.0463
X-F	0.5036	−0.6245	−0.6676	0.4076	−0.2500	0.5390	0.0437
X-Cl	0.5037	−0.6226	−0.6675	0.4081	−0.2503	0.5380	0.0450
X-Br	0.5037	−0.6221	−0.6717	0.4081	−0.2503	0.5379	0.0454
X-OCH_3_	0.5032	−0.6314	−0.6720	0.4054	−0.2518	0.5489	0.0404
X-OCH_2_CH_3_	0.5032	−0.6318	−0.6704	0.4051	−0.2515	0.5490	0.0402
X-CH_3_	0.5033	−0.6262	−0.6706	0.4064	−0.2484	0.5408	0.0442
X-CH_2_CH_3_	0.5031	−0.6253	−0.6708	0.4064	−0.2533	0.5503	0.0453
X-C(CH_3_)_3_	0.5032	−0.6252	−0.6645	0.4045	−0.2445	0.5495	0.0456
X-CN	0.5039	−0.6155	−0.6735	0.4105	−0.2574	0.5428	0.0490
X-NH_2_	0.5033	−0.6373	−0.6634	0.4034	−0.2443	0.5379	0.0362
X-NO_2_	0.5041	−0.6141	−0.6749	0.4111	−0.2531	0.5325	0.0493
X-N(CH_3_)_2_	0.5031	−0.6394	−0.6675	0.4027	−0.2475	0.5462	0.0355
X-COCCH_2_CH_3_	0.5033	−0.6197	−0.6710	0.4089	−0.2562	0.5464	0.0478
X-OH	0.5034	−0.6317	−0.6658	0.4055	−0.2473	0.5393	0.0393
Y-F	0.5034	−0.5997	−0.6651	0.4103	−0.2538	0.5452	0.0661
Y-Cl	0.5033	−0.6057	−0.6647	0.4117	−0.2569	0.5507	0.0593
Y-Br	0.5033	−0.6038	−0.6695	0.4115	−0.2575	0.5525	0.0609
Y-OCH_3_	0.5029	−0.6156	−0.6701	0.4092	−0.2502	0.5561	0.0539
Y-OCH_2_CH_3_	0.5027	−0.6161	−0.6683	0.4089	−0.2502	0.5576	0.0539
Y-CH_3_	0.5028	−0.6210	−0.6682	0.4091	−0.2585	0.5495	0.0473
Y-CH_2_CH_3_	0.5027	−0.6204	−0.6664	0.4095	−0.2604	0.5518	0.0478
Y-C(CH_3_)_3_	0.5027	−0.6213	−0.6609	0.4080	−0.2638	0.5524	0.0450
Y-CN	0.5049	−0.5951	−0.6722	0.4145	−0.2582	0.5364	0.0658
Y-NH_2_	0.5043	−0.6724	−0.6627	0.4039	−0.2438	0.5473	−0.0002
Y-NO_2_	0.5042	−0.6018	−0.6708	0.4138	−0.2567	0.5517	0.0610
Y-N(CH_3_)_2_	0.5024	−0.6287	−0.6634	0.4055	−0.2613	0.5475	0.0422
Y-COCCH_2_CH_3_	0.5043	−0.6037	−0.6685	0.4102	−0.2590	0.5321	0.0596
Y-OH	0.5063	−0.6902	−0.6682	0.4061	−0.2421	0.5447	−0.0217

## Data Availability

The data presented in this study are available in the Supplementary Materials.

## Supplementary Materials

The following supporting information can be downloaded at: https://www.mdpi.com/article/10.3390/molecules28135017/s1.
