# Supplementary material for: Effect of Substituent Groups on the Strength of Intramolecular Hydrogen Bonds in 2,4-Dihydroxybenzophenone UV Absorbers
Source: Molecules. 2023 Jun 27;28(13):5017. doi: 10.3390/molecules28135017 (PMC10343706; doi:10.3390/molecules28135017)
Supplement: Supplementary file 1 [file molecules-28-05017-s001.zip › molecules-2429582-supplementary.pdf]

Supporting Information

Effect of Substituent Groups on the Strength of Intramolecular Hydrogen Bonds in 2,4-dihydroxyb，Enzophenone UV Absorbers

Zhengjun Fang*, Xinhua Zhang, Feng Wu, Baoyu Huang*, Chaktong Au, Bing Yi*

Hunan Provincial Key Laboratory of Environmental Catalysis & Waste Recycling, College of Materials and Chemical Engineering, Hunan Institute of Engineering, Xiangtan 411104, China

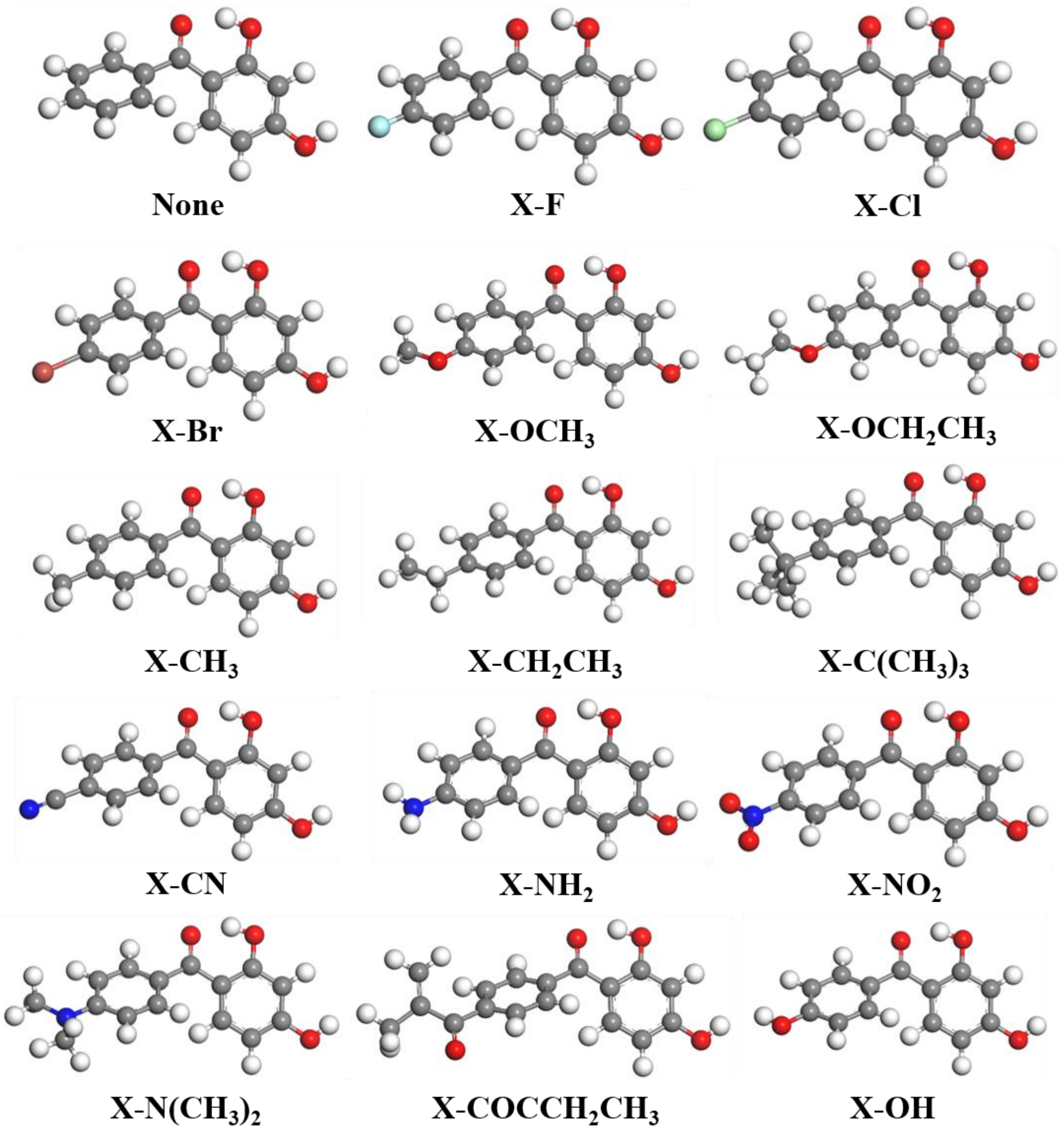

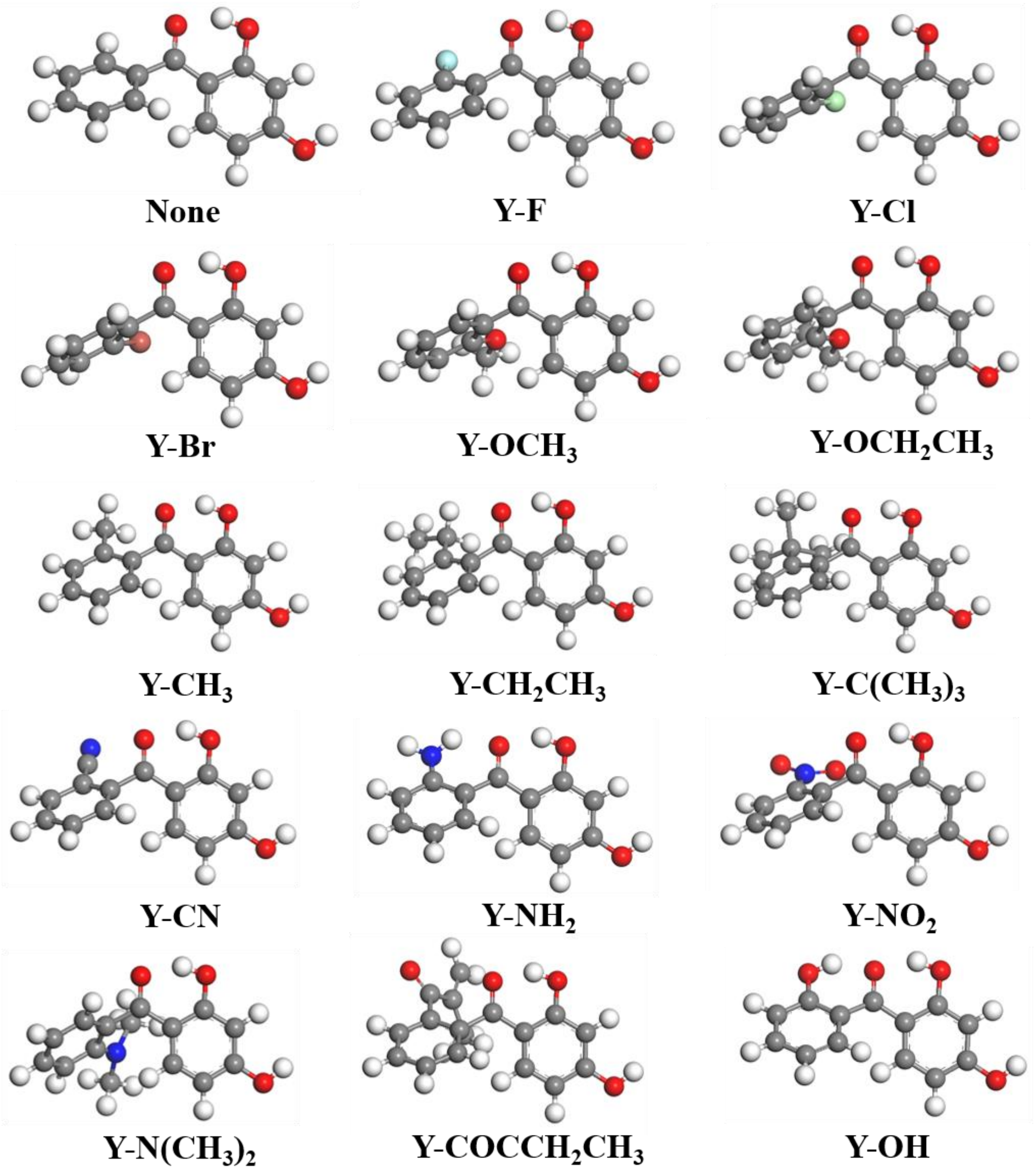

Figure S1 All the molecules optimized for stable structure involved in this study

Table S1 Experimental ultraviolet absorption spectra of 9 UV absorber molecules at wavelengths (nm)

	Experiment	
	(1)	(2)
None	287	327
X-F	292	324
X-Cl	292	324
X-Br	292	324
$X\text{-}OCH_3$	293	329
$X\text{-}OCH_2CH_3$	296	328
$X\text{-}CH_3$	292	321
$X\text{-}CH_2CH_3$	292	324
$X\text{-}C(CH_3)_3$	291	324

(1) represents the wavelength corresponding to the first absorption peak, (2) represents the wavelength corresponding to the second absorption peak

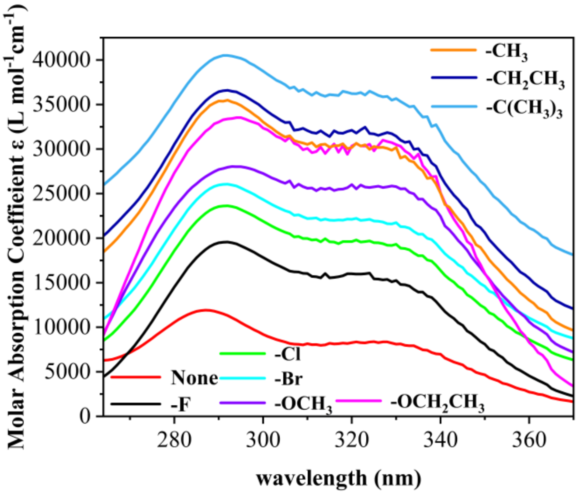

Figure S2 9 spectra of experimental measurements

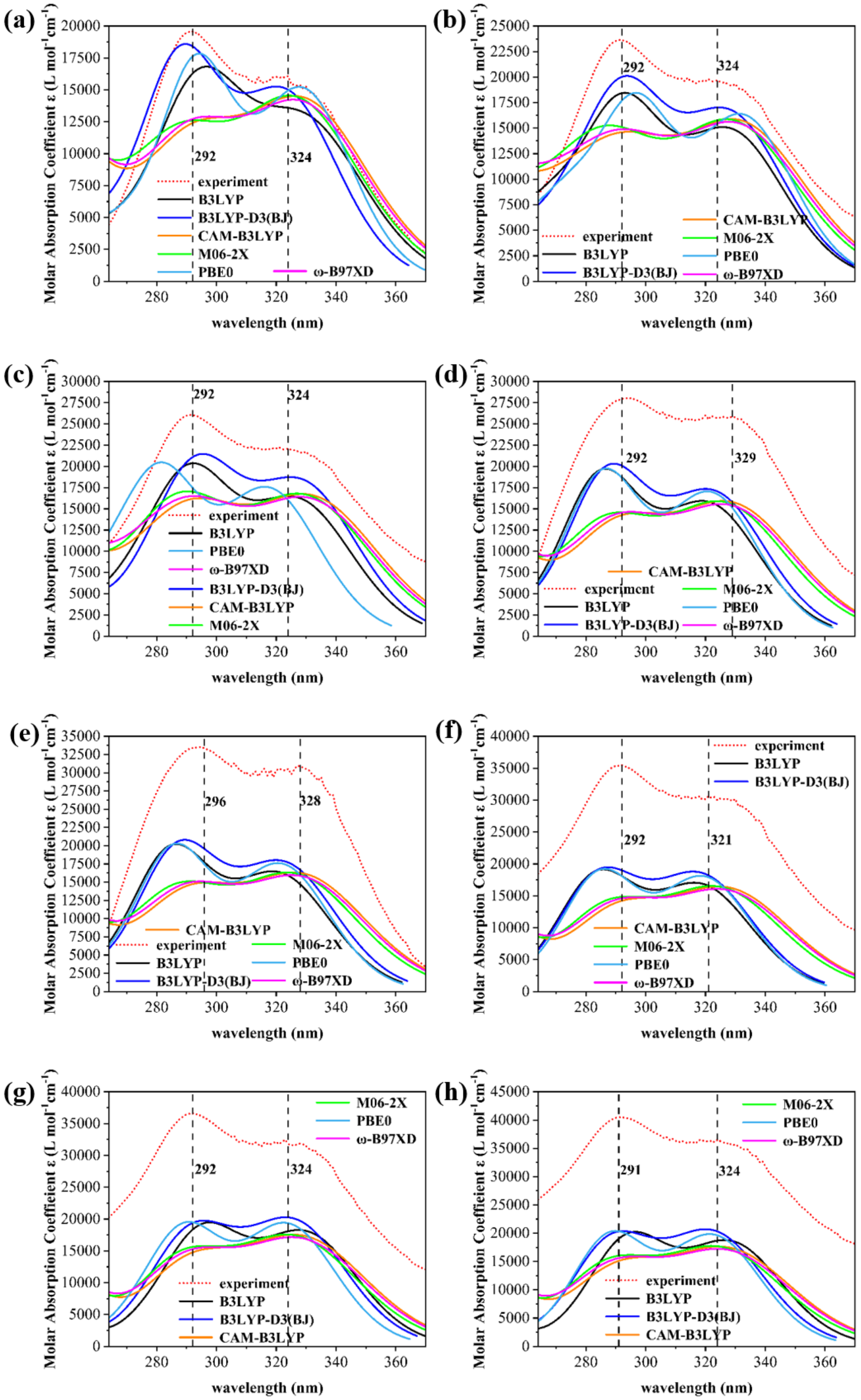

Figure S3 Spectra of 8 compounds calculated by 6 theoretical methods

(a) UV absorption spectra for the experimental and theoretical comparison of X-F, (b) UV absorption spectra for the experimental and theoretical comparison of X-Cl, (c) UV absorption spectra for the experimental and theoretical comparison of X-Br, (d) UV absorption spectra for the experimental and theoretical comparison of X-OCH_3, (e) UV absorption spectra for the experimental and theoretical comparison of X-OCH_2CH_3, (f) UV absorption spectra for the experimental and theoretical comparison of X-CH_3,(g) UV absorption spectra for the experimental and theoretical comparison of X-CH_2CH_3, (h) UV absorption spectra for the experimental and theoretical comparison of X-$C(CH_3)_3$.

Table S2 Contribution of 29 molecular leaps to the absorption peak

**Substituent**	(1)	The transitions that generate (1) and the amount of contribution	(2)	The transitions that generate (2) and the amount of contribution
None	290.0634	S0-S3(87.406), S0-S5(7.456)	326.4899	S0-S2(97.811)
X-F	290.364	S0-S3(87.492), S0-S5(6.568)	327.1589	S0-S2(97.415)
X-Cl	288.0866	S0-S3(69.476), S0-S5(22.615), S0-S4(7.410)	329.8316	S0-S2(97.209)
X-Br	290.2543	S0-S3(64.518), S0-S5(17.551), S0-S4(17.485)	327.1718	S0-S2(96.665)
X-OCH_3	291.3815	S0-S3(79.545), S0-S5(10.339), S0-S4(10.339)	323.8782	S0-S2(96.907)
X-OCH_2CH_3	289.2911	S0-S3(78.133), S0-S5(10.584), S0-S4(10.411)	326.8489	S0-S2(96.681)
X-CH_3	291.5552	S0-S3(77.997), S0-S5(12.475), S0-S4(8.250)	323.6799	S0-S2(96.817)

X-CH_2CH_3	292.3499	S0-S3(75.918), S0-S5(16.850), S0-S4(5.929)	325.6392	S0-S2(96.552)
X-$C(CH_3)_3$	291.1577	S0-S3(76.386), S0-S5(11.931), S0-S4(10.394)	324.3072	S0-S2(96.477)
X-CN	299.4641	S0-S3(95.996)	337.4549	S0-S2(97.824)
X-NH_2	284.8312	S0-S5(94.278), S0-S4(5.284)	338.9753	S0-S1(56.166), S0-S2(43.645)
X-NO_2	286.1476	S0-S7(75.831), S0-S8(24.031)	342.4935	S0-S5(86.819), S0-S6(12.204)
X-$N(CH_3)_2$	285.1543	S0-S5(95.232)	358.2394	S0-S1(97.009)
X-$COCCH_2CH_3$	297.6737	S0-S6(61.965), S0-S4(23.718), S0-S8(7.841), S0-S5(5.379)	334.4127	S0-S3(96.273)
X-OH	296.4882	S0-S3(83.154), S0-S4(14.568)	328.7072	S0-S2(94.082), S0-S1(5.444)
Y-F	290.8454	S0-S3(92.618)	326.4899	S0-S2(97.032)
Y-Cl	293.4198	S0-S3(91.361)	322.2043	S0-S2(95.771)
Y-Br	293.6878	S0-S3(88.228), S0-S4(7.458)	322.45	S0-S2(95.719)
Y-OCH_3	289.6093	S0-S4(94.937)	321.5068	S0-S2(91.256)
Y-OCH_2CH_3	289.2191	S0-S4(94.894)	324.8325	S0-S2(91.139)
Y-CH_3	291.1949	S0-S3(67.220), S0-S4(22.716), S0-S5(7.865)	324.4783	S0-S2(97.035)

Y-CH_2CH_3	290.3908	S0-S3(62.259), S0-S4(29.637), S0-S5(5.966)	322.7733	S0-S2(97.071)
Y-$C(CH_3)_3$	290.0325	S0-S3(87.205), S0-S4(6.840)	321.6817	S0-S2(97.636)
Y-CN	291.9237	S0-S3(89.653), S0-S4(6.260)	317.8255	S0-S2(97.737)
Y-NH_2	295.2085	S0-S4(89.462) S0-S5(7.383)	326.9927	S0-S3(97.796)
Y-NO_2	283.3391	S0-S7(55.161), S0-S8(25.941), S0-S9(10.225), S0-S6(7.5027)	330.2887	S0-S4(90.652)
Y-$N(CH_3)_2$	272.2307	S0-S7(46.261), S0-S6(40.027), S0-S5(11.439)	316.7152	S0-S3(95.498)
Y-$COCCH_2CH_3$	290.7826	S0-S7(49.992), S0-S6(25.726), S0-S5(13.450)	323.9911	S0-S3(97.787)
Y-OH	295.0651	S0-S4(86.265), S0-S5(13.557)	331.2789	S0-S1(98.646)

Only transitions with a contribution greater than 5% are counted. (1) represents the wavelength corresponding to the first absorption peak, (2) represents the wavelength corresponding to the second absorption peak

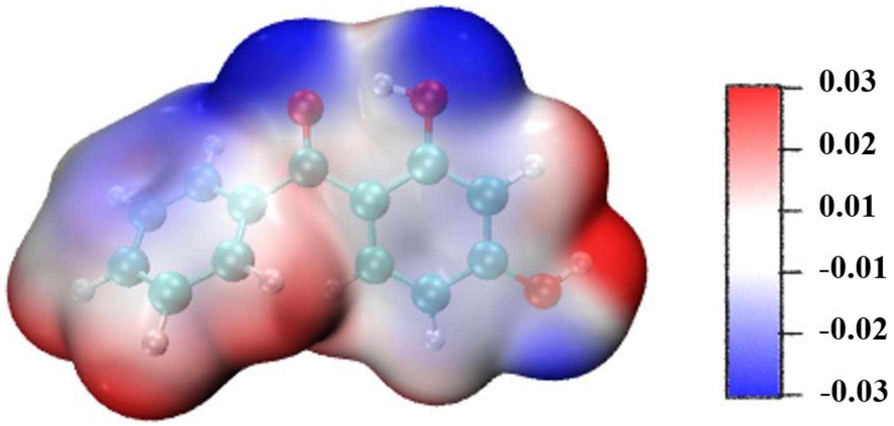

Figure S4 shows the electrostatic potential of 2,4-DBH at the Y substitution position. The units are a.u.

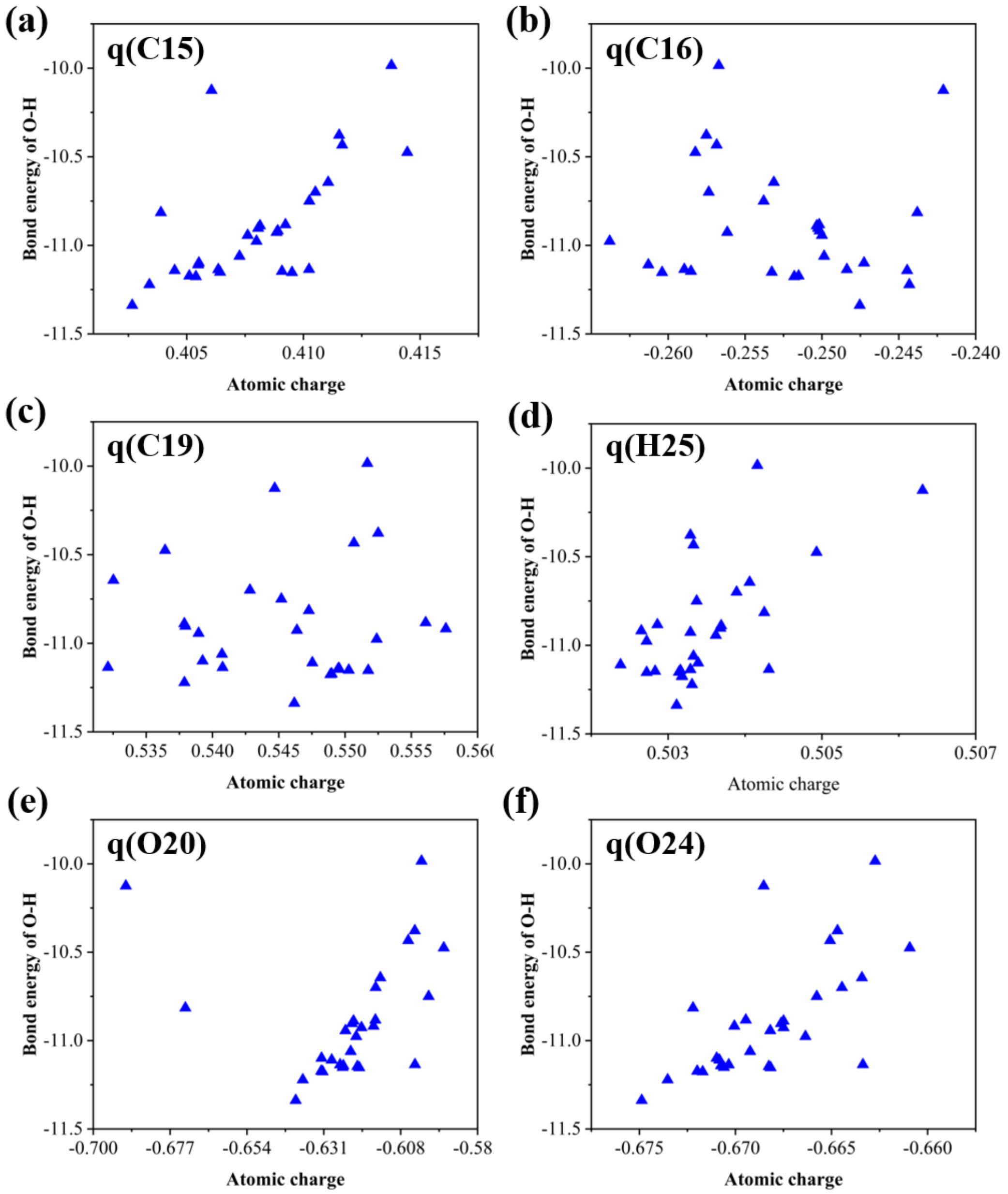

Figure S5 Diagram of the bond energy of individual atoms in the chelate ring in relation to the hydrogen bond
